# A novel role for Hsc70–4 in blood cell differentiation in *Drosophila*

**DOI:** 10.3389/fimmu.2025.1641695

**Published:** 2025-10-08

**Authors:** Bayan Kharrat, Erika Gábor, Péter Vilmos, Lauren M. Goins, Viktor Honti

**Affiliations:** ^1^ Drosophila Blood Cell Differentiation Group, Institute of Genetics, HUN-REN Biological Research Centre, Szeged, Hungary; ^2^ Department of Developmental Biology, Stanford University School of Medicine, Stanford, CA, United States; ^3^ Drosophila Nuclear Actin Laboratory, Institute of Genetics, HUN-REN Biological Research Centre, Szeged, Hungary

**Keywords:** Hsc70-4, hematopoiesis, lamellocytes, blood cell, *Drosophila*

## Abstract

The *Drosophila* lymph gland serves as an excellent model for studying blood cell development, closely mirroring the key components of mammalian hematopoietic niches: blood cell progenitors, mature blood cells, and niche cells that secrete signals to regulate progenitor maintenance. In the *Drosophila* larva, two primary types of mature hemocytes exist: macrophage-like plasmatocytes and platelet-like crystal cells. In cases of immune challenge or neoplastic conditions, a third type of hemocyte, the lamellocyte, appears to encapsulate large invaders that plasmatocytes cannot phagocytose. Importantly, the spontaneous appearance of lamellocytes in unchallenged larvae indicates defects in progenitor maintenance or blood cell fate regulation. In this study, we uncover a novel role for the molecular chaperone Hsc70–4 in suppressing lamellocyte differentiation across all three domains of the lymph gland. We show that Hsc70–4 depletion in the niche induces non-apoptotic cell death and oxidative stress, which in turn drives non-cell-autonomous lamellocyte differentiation via the Akt/Foxo pathway. In blood cell progenitors, particularly distal progenitors, Hsc70–4 loss promotes cell-autonomous lamellocyte differentiation, thereby diminishing the progenitor pool. Furthermore, silencing *Hsc70–4* in mature hemocytes elicits a strong immune response characterized by primary lobe disintegration, lamellocyte transdifferentiation, and melanotic tumor formation. Together, these findings highlight the multifaceted roles of Hsc70–4 in *Drosophila* hematopoiesis, offering valuable insights that could enhance our understanding of the role of its orthologue in mammals and humans.

## 1 Introduction

Hematopoietic stem cells (HSCs) and blood cell precursors are maintained by intricate cell-autonomous and non-cell-autonomous signaling networks across the evolutionary spectrum, with numerous conserved mechanisms between *Drosophila* and humans (1–4). In *Drosophila*, the lymph gland, a multilobed hematopoietic organ, serves as a major source of larval blood cells (hemocytes). Within the primary (anterior) lobes of the lymph gland, the medullary zone (MZ) contains progenitors that differentiate into mature hemocytes that populate the cortical zone (CZ) of the lobe. At the posterior end of the primary lobes, the lymph gland harbors a hematopoietic niche (the posterior signaling center (PSC)), which emits signals to the MZ to regulate progenitor differentiation (2, 5–8). Two types of differentiated hemocytes can be distinguished in the CZ and the larval circulation: phagocytic plasmatocytes and melanin-producing cells, known as crystal cells (9–11). Immune challenges or defects in progenitor maintenance signals can trigger the differentiation of larger, specialized cells called lamellocytes. The primary function of lamellocytes is to encapsulate larger particles that plasmatocytes are unable to engulf, such as parasitic wasp eggs (9, 12–16). Because lamellocytes are absent under uninduced conditions, their emergence in naive animals may suggest a malfunction in the mechanisms maintaining hemocyte progenitors in the larva (17, 18).

Heat shock proteins (Hsps) in *Drosophila* are a conserved family of molecular chaperones that maintain protein homeostasis by assisting in folding, stabilization, and degradation of proteins under both physiological and stress conditions (19, 20). Although traditionally associated with stress responses, many Hsps, such as the Heat Shock Cognate 70 (Hsc70) proteins, are constitutively expressed and carry out essential housekeeping functions (21). In a genetic screen for novel regulators of hemocyte differentiation, we identified Heat shock protein 70 cognate 4 (Hsc70-4) as a gene of interest. Hsc70–4 is the best-studied *Drosophila* Hsc70 member, and it is involved in diverse cellular processes, including maintenance of neuromuscular junctions (21), germline stem cell differentiation (22), and eye development (23). Within the hematopoietic system, Hsc70–4 is expressed in both the lymph gland and circulating hemocytes, and prior studies have found that *Hsc70–4* mutants develop melanotic tumors in their hemocoel (24). Additionally, Hsc70–4 has been reported to be part of a chaperonin complex together with Mlf and DNAj-1, which stabilizes Lozenge (Lz), a transcription factor crucial for specifying crystal cell fate in *Drosophila* cell lines (25, 26). Despite these findings, the function of Hsc70–4 in the different lymph gland zones, and its influence on other effector hemocytes and progenitors, remain poorly understood.

In this paper, we report a previously uncharacterized role of the molecular chaperone Hsc70–4 in suppressing lamellocyte differentiation throughout all three domains of the lymph gland. Our findings demonstrate that Hsc70–4 supports progenitor maintenance via both cell-autonomous and non-cell-autonomous mechanisms. Additionally, we show its involvement in the transdifferentiation of plasmatocytes into lamellocytes in the lymph gland and circulation. Notably, silencing *Hsc70–4* in differentiated hemocytes triggers primary lobe disintegration and melanotic tumor formation. Together, these findings reveal context-dependent functions of Hsc70–4 and highlight its central role in regulating *Drosophila* hematopoiesis.

## 2 Results

### 2.1 Hsc70–4 controls hematopoietic niche size and function in the lymph gland

**Figure 1:**
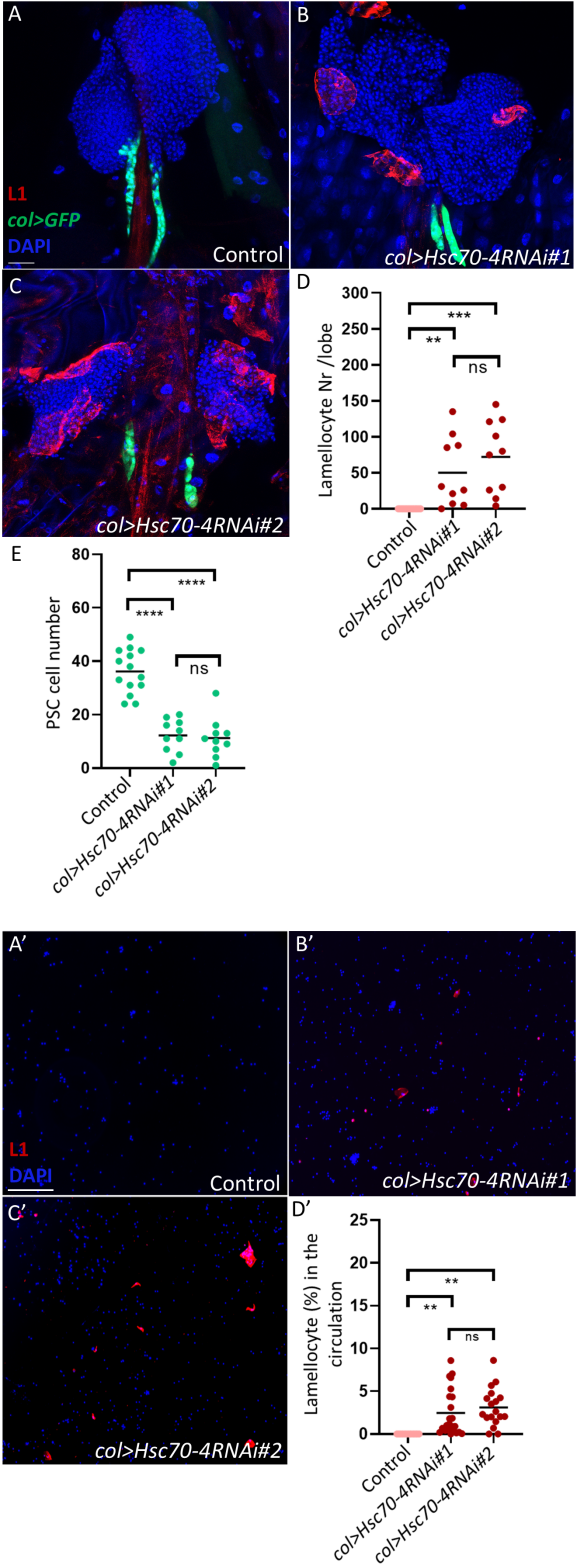
Hsc70–4 controls PSC size and function. **(A-C)** Silencing *Hsc70–4* using 2 different RNAi lines (*Pcol85-Gal4,UAS-2xEGFP/+; UAS-Hsc70-4RNAi#1/+*) (*n =* 10) **(B)** (*Pcol85-Gal4,UAS-2xEGFP/+; UAS-Hsc70-4RNAi#2/+*) (*n =* 10) **(C)** causes lamellocyte differentiation and decrease in the niche size in comparison to the control (*Pcol85-Gal4,UAS-2xEGFP/+*) (*n =* 14) **(A)** (blue: nuclei, green: PSC, red: lamellocytes). *n* refers to the number of lymph gland lobes analyzed. Scale bar: 20 μm. **(D)** A scatter dot plot showing the number of lamellocytes per lymph gland lobe in the genotypes presented in panels **(A-C)**. Each dot on the graph represents one lymph gland lobe. Data were analyzed using ANOVA with Tukey’s test for multiple comparisons, ***p ≤ 0.001, **p ≤ 0.01, *ns*, non-significant. **(E)** A scatter dot plot showing PSC cell number in larvae from the genotypes presented in panels **(A-C)**. Each dot on the graph represents a PSC from one lymph gland lobe. Data were analyzed using ANOVA with Tukey’s test for multiple comparisons, ****p ≤ 0.0001, *ns*, non-significant. (A’-C’) Unlike in the control where lamellocytes are not detected (*n =* 12) **(A’)**, lamellocytes appear in the circulation when *Hsc70–4* is silenced using two different RNAi lines (*Pcol85-Gal4,UAS-2xEGFP/+; UAS-Hsc70-4RNAi#1/+*) (*n* = 23) **(B’)** (*Pcol85-Gal4,UAS-2xEGFP/+; UAS-Hsc70-4RNAi#2/+*) (*n* = 18) **(C’)** (blue: nuclei, red: lamellocytes). *n* refers to the number of larvae analyzed. Scale bar: 20 μm. **(D’)** A scatter dot plot showing the percentage of lamellocytes in the circulation of larvae from the genotypes presented in panels **(A’-C’)**. Each dot on the graph represents a single larva. Data were analyzed using ANOVA with Tukey’s test for multiple comparisons, **p ≤ 0.01, *ns*, non-significant.

In a screen aimed to identify genes whose silencing triggers lamellocyte differentiation in the larva, we identified *Hsc70-4*. Using two independent RNAi lines (*Hsc70-4RNAi#1* [BDSC#28709] and *Hsc70-4RNAi#2* [BDSC#35684]), we found that PSC-specific silencing of *Hsc70–4* in the lymph gland, driven by *col-Gal4*, induces the differentiation of lamellocytes in the larval hematopoietic organ and their subsequent appearance in circulation (**Figures 1A-C, A’-C’**, quantification in **Figures 1D, D’**). This suggests that *Hsc70–4* is required in the hematopoietic niche to suppress progenitor differentiation in a non-cell-autonomous manner.

Further analysis of *Hsc70-4RNAi#1* (hereafter referred to as *Hsc70-4RNAi*) lymph glands revealed that silencing *Hsc70–4* in the PSC also reduces crystal cell differentiation in the lymph gland, without significantly affecting plasmatocyte differentiation (**Supplementary Figures S1A, B, D, E**, quantification in **Supplementary Figures S1C, F**). This observation is consistent with previous experiments by our group and others suggesting that when lamellocyte differentiation is triggered in the lymph gland, the fate of crystal cells is less favored (27, 28).

As niche size has been shown to correlate with progenitor differentiation rates (29), we investigated whether silencing *Hsc70–4* impacts niche cell number. We found that both *Hsc70-4RNAi* lines caused a significant reduction in niche size compared to the control (**Figures 1A–C**, quantification in **Figure 1E**). Importantly, manipulation of *Hsc70–4* with an alternative PSC driver, *Antp-GAL4*, also decreased niche size (**Supplementary Figures S1G, H**, quantification in **Supplementary Figure S1I**), further reinforcing that Hsc70–4 is essential for maintaining normal hematopoietic niche size and function in the lymph gland.

### 2.2 Hsc70–4 depletion in the niche causes non-apoptotic cell death of niche cells

**Figure 2:**
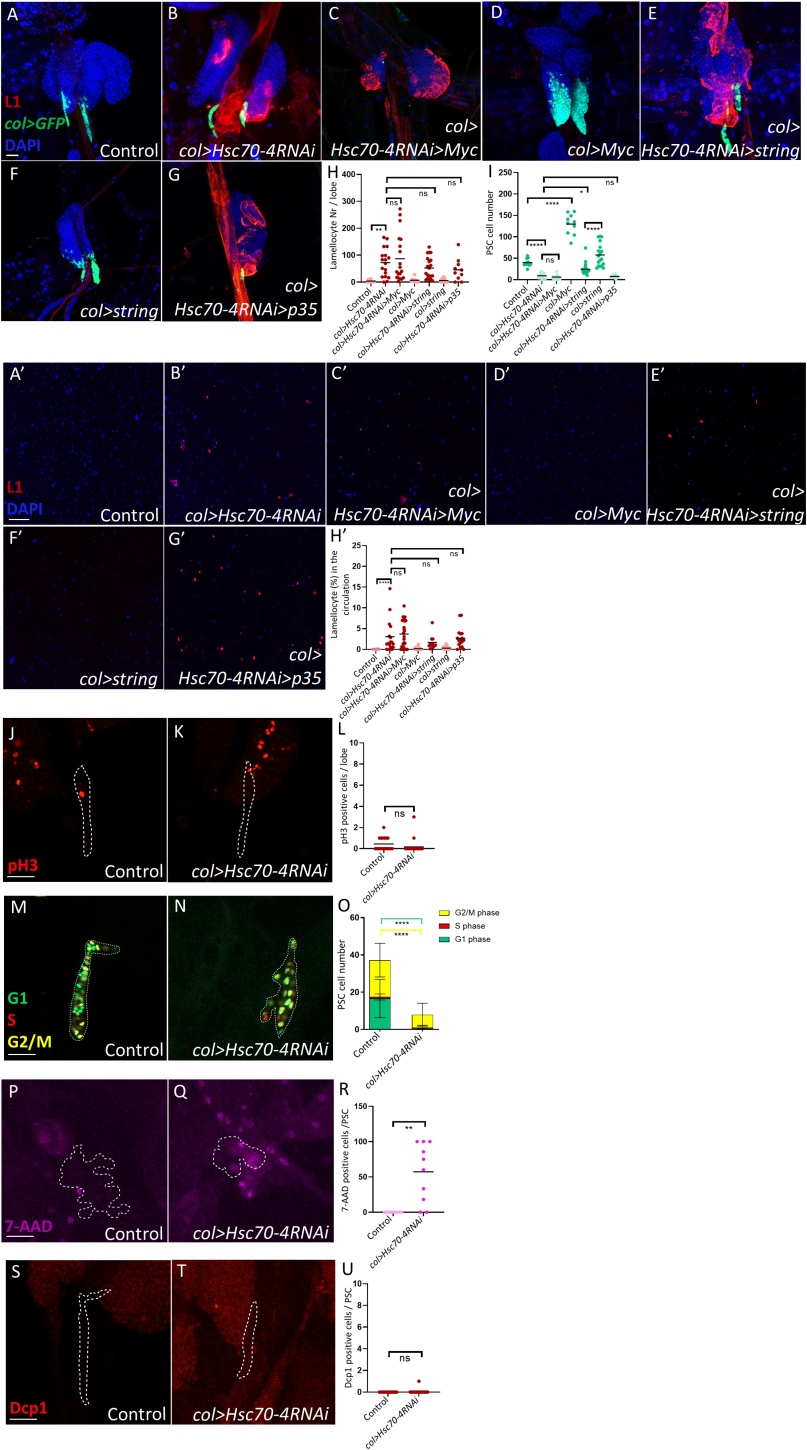
Hsc70–4 causes non-apoptotic niche cell death. **(A, B)** Silencing *Hsc70-4* (*Pcol85-Gal4,UAS-2xEGFP/+; UAS-Hsc70-4RNAi/+*) (*n =* 18) **(B)** results in lamellocyte differentiation and a reduction in niche size compared to the control (*Pcol85-Gal4,UAS-2xEGFP/+*) (*n =* 12) **(A)**. **(C, D)** Overexpression of *Myc* fails to restore the reduced PSC size and lamellocyte differentiation observed in *col>Hsc70-4RNAi* animals (*Pcol85-Gal4,UAS-2xEGFP/UAS-Myc; UAS-Hsc70-4RNAi/+*) (*n =* 18) **(C)**, whereas it significantly increases niche size in the control group (*Pcol85-Gal4,UAS-2xEGFP/UAS-Myc*) (*n =* 10) **(D)**. **(E, F)** Overexpression of *string* increases the niche size in *col>Hsc70-4RNAi* larvae without rescuing lamellocyte differentiation in these animals (*Pcol85-Gal4,UAS-2xEGFP/UAS-string; UAS-Hsc70-4RNAi/+*) (*n =* 20) **(E)**. However, the number of niche cells remains lower compared to larvae where *string* is overexpressed alone (*Pcol85-Gal4,UAS-2xEGFP/UAS-string*) (*n =* 18) **(F)**. **(G)** Suppression of apoptosis via *p35* overexpression does not rescue the reduced niche size or lamellocyte differentiation observed in *col>Hsc70-4RNAi* larvae (*n =* 10) (blue: nuclei, green: PSC, red: lamellocytes). *n* refers to the number of lymph gland lobes analyzed. Scale bar: 20 μm. **(H)** A scatter dot plot illustrating the number of lamellocytes per lymph gland lobe across the genotypes shown in panels **(A-G)**. Each dot represents one lymph gland lobe. Data were analyzed using ANOVA with Tukey’s multiple comparisons test, **p ≤ 0.01, *ns*, not significant. **(I)** A scatter dot plot showing PSC cell numbers in larvae with the genotypes presented in panels **(A-G)**. Each dot represents the PSCs from a single lymph gland lobe. Data were analyzed using ANOVA with Tukey’s multiple comparisons test, *p ≤ 0.05, ****p ≤ 0.0001, *ns*, not significant. **(A’, B’)** Silencing *Hsc70-4* (*Pcol85-Gal4,UAS-2xEGFP/+; UAS-Hsc70-4RNAi/+*) (*n =* 17) **(B’)** results in the appearance of lamellocytes in the circulation, which is not observed in the control (*Pcol85-Gal4,UAS-2xEGFP/+*) (*n =* 24) **(A’)**. **(C’, D’)** Overexpression of *Myc* fails to rescue lamellocyte appearance observed in the circulation of *col>Hsc70-4RNAi* animals (*Pcol85-Gal4,UAS-2xEGFP/UAS-Myc; UAS-Hsc70-4RNAi/+*) (*n =* 27) **(C’)**, and overexpression of *Myc* alone in the niche does not cause lamellocyte differentiation in the circulation (*Pcol85-Gal4,UAS-2xEGFP/UAS-Myc*) (*n =* 36) **(D’)**. **(E’, F’)** Overexpression of *string* also fails to rescue lamellocyte appearance observed in the circulation of *col>Hsc70-4RNAi* larvae (*Pcol85-Gal4,UAS-2xEGFP/UAS-string; UAS-Hsc70-4RNAi/+*) (*n =* 11) **(E’)**, and overexpression of *string* alone in the niche does not cause lamellocyte differentiation in the circulation (*Pcol85-Gal4,UAS-2xEGFP/UAS-string*) (*n =* 48) **(F’)**. **(G’)** Suppression of apoptosis via *p35* overexpression does not rescue lamellocyte appearance observed in *col>Hsc70-4RNAi* larvae (*n =* 17) (blue: nuclei, red: lamellocytes). *n* refers to the number of larvae analyzed. Scale bar: 20 μm. **(H’)** A scatter dot plot showing the percentage of lamellocytes in the circulation of larvae from the genotypes presented in panels **(A’-G’)**. Each dot on the graph represents a single larva. Data were analyzed using ANOVA with Tukey’s test for multiple comparisons, ****p ≤ 0.0001, *ns*, not significant. **(J, K)** No significant difference is observed in the number of dividing cells (pH3 positive, red) between *col>Hsc70-4RNAi* niches (outlined by dashed lines, based on *col>GFP* expression) (*Pcol85-Gal4,UAS-2xEGFP/+; UAS-Hsc70-4RNAi/+*) (*n* = 24) **(K)** and the control (*Pcol85-Gal4,UAS-2xEGFP/+*) (*n* = 18) **(J)**. *n* refers to the number of lymph gland lobes analyzed. Scale bar: 20 μm. **(L)** A scatter dot plot quantifying the number of pH3 positive cells in the niches (*col>GFP* positive cells) of genotypes presented in the panels **(J, K)**. Each dot on the graph represents a PSC from one lymph gland lobe. Data were analyzed using two-tailed unpaired Student’s t-test, *ns*, not significant. **(M, N)** The FUCCI cell cycle reporter pattern (green: G1 phase, red: S phase, yellow: G2/M phase) in the niche (outlined by dashed lines, based on *col>GFP* expression) of *col>Hsc70-4RNAi* (*Pcol85-Gal4/+; UAS-Hsc70-4RNAi/UAS-EGFP::E2F1^1-230,^UAS-mRFP1::CycB^1-266^*) (*n =* 14) **(N)** compared to the control (*Pcol85-Gal4/+; UAS-EGFP::E2F1^1-230,^UAS-mRFP1::CycB^1-266^*) (*n =* 16) **(M)**. *n* refers to the number of lymph gland lobes analyzed. Scale bar: 20 μm. **(O)** A bar graph showing the mean and standard deviation of the number of niche cells in the G1, S, and G2/M phase in the genotypes presented in the panels **(M, N)**. Data were analyzed using ANOVA with Tukey’s test for multiple comparisons, ****p ≤ 0.0001. **(P, Q)** An increase in the number of dead or dying cells (7-AAD positive, magenta) is observed when *Hsc70–4* is silenced in the niche (outlined by dashed lines, based on *col>GFP* expression) (*Pcol85-Gal4,UAS-2xEGFP/+; UAS-Hsc70-4RNAi/+*) (*n =* 10) **(Q)** in comparison to the control (*Pcol85-Gal4,UAS-2xEGFP/+*) (*n =* 8) **(P)**. *n* refers to the number of lymph gland lobes analyzed. Scale bar: 20 μm. **(R)** A scatter dot plot quantifying the number of 7-AAD positive cells in the niches (*col>GFP* positive cells) of genotypes presented in the panels **(P, Q)**. Each dot in the graph represents a PSC from one lymph gland lobe. Data were analyzed using two-tailed unpaired Student’s t-test, **p ≤ 0.01. **(S, T)** No significant difference is observed in the number of apoptotic cells (Dcp1 positive, red) when *Hsc70–4* is silenced in the niche (outlined by dashed lines, based on *col>GFP* expression) (*Pcol85-Gal4,UAS-2xEGFP/+; UAS-Hsc70-4RNAi/+*) (*n =* 14) **(T)** in comparison to the control (*Pcol85-Gal4,UAS-2xEGFP/+*) (*n =* 12) **(S)**. *n* refers to the number of lymph gland lobes analyzed. Scale bar: 20 μm. **(U)** A scatter dot plot quantifying the number of Dcp1 positive cells in the niches (*col>GFP* positive cells) of genotypes presented in the panels **(S, T)**. Each dot in the graph represents a PSC from one lymph gland lobe. Data were analyzed using two-tailed unpaired Student’s t-test, *ns*, not significant.

We next sought to determine the cause of the reduced niche size observed in *col>Hsc70-4RNAi* lymph glands. Previous studies have shown that the Myc oncoprotein promotes niche growth downstream of Dpp and Wg signaling by stimulating cell proliferation (29). To test whether Hsc70–4 depletion reduces niche size by suppressing Myc-driven cell division, we overexpressed *Myc* in *col>Hsc70-4RNAi* larvae. However, Myc overexpression failed to rescue either the reduction in niche size or the appearance of lamellocytes in the lymph gland and circulation (**Figures 2A-D, A’-D’**, quantification in **Figures 2H, I, H’**). Consistent with this, phospho-histone H3 (pH3) staining, which marks mitotic cells (30) showed no significant difference between *col>Hsc70-4RNAi* niches and controls (**Figures 2J, K**, quantification in **Figure 2L**). These results indicate that the smaller niche phenotype in *col>Hsc70-4RNAi* larvae is not due to impaired cell division.

To further assess whether Hsc70–4 silencing disrupts normal cell cycle progression, we used the FUCCI system to examine the cell cycle profile in the niche (31). Notably, most remaining PSC cells in *col>Hsc70-4RNAi* animals were in the G2/M phase, with a marked loss of cells in the G1 phase (**Figures 2M, N**, quantification in **Figure 2O**). To exclude the possibility that G2/M stalling in *col>Hsc70-4RNAi* niches accounts for the reduced niche size, we overexpressed String (Cdc25), a kinase that promotes progression through the G2 phase (32, 33). While this intervention modestly increased PSC cell number in *col>Hsc70-4RNAi* larvae, the PSC size remained smaller than in control *col>string* animals, and lamellocyte differentiation was not rescued (**Figures 2E, F, E’, F’**, quantification in **Figures 2H, I, H’**).

Collectively, these findings suggest that the reduction in niche size following Hsc70–4 depletion is not attributable to decreased cell division or a slower cell cycle. Instead, the most plausible explanation is that Hsc70–4 depletion induces niche cell death, with cells in the G1 phase being particularly vulnerable. Indeed, 7-AAD staining, which labels dead or dying cells (34), was frequently observed in the PSCs of *col>Hsc70-4RNAi* animals but was absent in controls (**Figures 2P, Q**, quantification in **Figure 2R**). In contrast, staining with the apoptosis marker Dcp-1 showed no significant increase in PSC apoptosis (**Figures 2S, T**, quantification in **Figure 2U**). Moreover, overexpression of the apoptosis inhibitor p35 did not rescue PSC size in *col>Hsc70-4RNAi* larvae (**Figures 2G, G’**, quantification in **Figures 2H, I, H’**).

Taken together, these results indicate that Hsc70–4 depletion reduces niche size not by impairing proliferation or causing apoptosis, but instead by inducing non-apoptotic cell death. Importantly, cells in the G1 phase appear to be particularly sensitive to Hsc70–4 loss.

### 2.3 Depletion of Hsc70–4 in the niche induces lamellocyte differentiation through elevating cellular stress in the PSC

**Figure 3:**
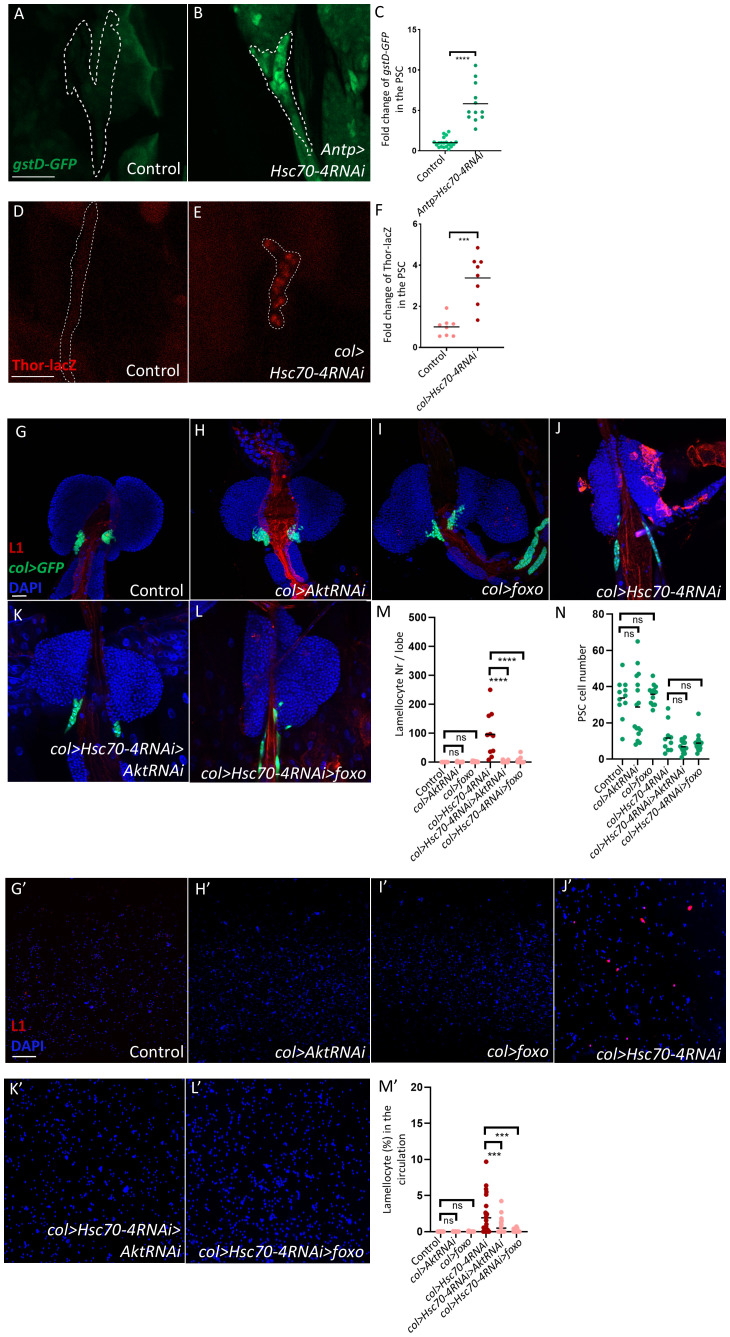
Silencing *Hsc70–4* in the PSC niche induces cellular stress and consequent appearance of lamellocytes in the lymph gland and circulation. **(A, B)** Silencing *Hsc70–4* induces high expression levels of the *gstD-GFP* reporter in the PSC (outlined by dashed lines, based on Col staining) (*gstD-GFP/+; Antp-Gal4/UAS-Hsc70-4RNAi*) (*n =* 12) **(B)**, in comparison to the control (*gstD-GFP/+; Antp-Gal4/+*) (*n =* 20) **(A)** (green: *gstD-GFP*). *n* indicates the number of lymph gland lobes examined. Scale bar: 20 μm. **(C)** A scatter dot plot showing the fold change (average = 5.8 folds) increase in the mean fluorescence intensity of *gstD-GFP* in the PSC upon silencing *Hsc70-4*. Each dot on the graph represents a PSC from a single lobe. Data were analyzed using two-tailed unpaired Student’s t-test, ****p ≤ 0.0001. **(D, E)** Silencing *Hsc70–4* triggers higher transcription of *Thor* in the niche (outlined by dashed lines, based on *col>GFP* expression), as revealed by anti-lacZ staining of the Thor-lacZ reporter (*Pcol85-Gal4,UAS-2xEGFP/Thor-lacZ; UAS-Hsc70-4RNAi/+*) (*n =* 8) **(E)** in comparison to the control (*Pcol85-Gal4,UAS-2xEGFP/Thor-lacZ*) (*n =* 8) **(D)** (red: Thor-LacZ). *n* indicates the number of lymph gland lobes examined. Scale bar: 20 μm. **(F)** A scatter dot plot showing the fold change (average = 3.3 folds) increase in the mean fluorescence intensity of Thor-LacZ in the PSC upon silencing *Hsc70-4*. Each dot on the graph represents a PSC from a single lobe. Data were analyzed using two-tailed unpaired Student’s t-test, ***p ≤ 0.001. **(G-I)** Silencing *Akt* (*Pcol85-Gal4,UAS-2xEGFP; +/UAS-AktRNAi*) (*n =* 16) **(H)** or overexpressing *foxo* (*Pcol85-Gal4,UAS-2xEGFP/UAS-foxo;+/+*) (*n =* 12) **(I)** does not lead to lamellocyte differentiation or affect PSC size in the control (*Pcol85-Gal4,UAS-2xEGFP/+; +/UAS-AktRNAi*) (*n =* 11) **(G)**. **(J-L)** Knocking down *Akt* (*Pcol85-Gal4,UAS-2xEGFP/+; UAS-Hsc70-4RNAi/UAS-AktRNAi*) (*n =* 16) **(K)** and overexpression of *foxo* (*Pcol85-Gal4,UAS-2xEGFP/UAS-foxo; UAS-Hsc70-4RNAi/+*) (*n =* 16) **(L)** rescue lamellocyte differentiation observed in *col>Hsc70-4RNAi* lymph glands (*Pcol85-Gal4,UAS-2xEGFP/+; UAS-Hsc70-4RNAi/+*) (*n =* 10) **(J)** (blue: nuclei, green: PSC, red: lamellocytes), with no effect on PSC size. *n* indicates the number of lymph gland lobes examined. Scale bar: 20 μm. **(M)** A scatter dot plot displaying the number of lamellocytes per lymph gland lobe for the genotypes shown in the panels **(G-L)**. Each dot on the graph represents a single lymph gland lobe. Data were analyzed using ANOVA with Tukey’s test for multiple comparisons, ****p ≤ 0.0001, *ns*, not significant. **(N)** A scatter dot plot displaying the number of PSC cells in the genotypes shown in the panels **(G-L)**. Each dot on the graph represents a PSC from a single lobe. Data were analyzed using ANOVA with Tukey’s test for multiple comparisons, *ns*, non-significant. **(G’-I’)** Similarly to the control (*Pcol85-Gal4,UAS-2xEGFP/+; +/+)* (*n =* 12) **(G’)**, silencing *Akt* (*Pcol85-Gal4,UAS-2xEGFP/+; +/UAS-AktRNAi*) (*n =* 12) **(H’)** or overexpressing *foxo* (*Pcol85-Gal4,UAS-2xEGFP/UAS-foxo;+/+*) (*n =* 12) **(I’)** does not lead to lamellocyte differentiation in the circulation. **(J’-L’)** knocking down *Akt* (*Pcol85-Gal4,UAS-2xEGFP/+; UAS-Hsc70-4RNAi/UAS-AktRNAi*) (*n =* 32) **(K’)** and overexpression of *foxo* (*Pcol85-Gal4,UAS-2xEGFP/UAS-foxo; UAS-Hsc70-4RNAi/+*) (*n =* 30) **(L’)** suppress lamellocyte numbers in the circulation of these larvae (*Pcol85-Gal4,UAS-2xEGFP/+; UAS-Hsc70-4RNAi/+*) (*n =* 29) **(J’)** (blue: nuclei, red: lamellocytes). *n* indicates the number of larvae examined. Scale bar: 20 μm. **(M’)** A scatter dot plot displaying the percentage of lamellocytes in the circulation of larvae from the genotypes shown in panels **(G’-L’)**. Each dot on the graph represents one single larva. Data were analyzed using ANOVA with Tukey’s test for multiple comparisons, ***p ≤ 0.001, *ns*, non-significant.

Since Hsc70–4 is a chaperone that maintains cellular homeostasis by promoting proper protein folding and preventing protein accumulation (35, 36), and because elevated ROS in the niche can non–cell-autonomously trigger lamellocyte differentiation (27, 37–39), we tested whether Hsc70–4 depletion leads to ROS accumulation in PSC cells. We found that silencing *Hsc70–4* using the PSC-specific drivers *Antp-Gal4* or *col-Gal4* led to an upregulation of the stress reporters *gstD-GFP* and *Thor-lacZ* (27, 39–41), respectively (**Figures 3A, B, D, E**, quantification in **Figures 3C, F**). The use of two independent drivers and two distinct ROS reporters strengthens the conclusion that Hsc70–4 is essential for maintaining redox homeostasis in the PSC.

Because the Akt/Foxo pathway has been implicated in oxidative sensing in the niche (39), we next asked whether modulating this pathway could suppress the lamellocyte phenotype in *col>Hsc70-4RNAi* animals. Indeed, silencing *Akt* or overexpressing *foxo* prevented lamellocyte appearance in both the lymph gland and circulation of these animals (**Figures 3G–L, G’–L’**, quantification in **Figures 3M, M’**). However, these manipulations did not reduce ROS accumulation in the niche of these larvae (**Supplementary Figures S2A–D**, quantification in **Supplementary Figure S2E**), suggesting that Akt/Foxo functions downstream to ROS in this context. This is consistent with previous work in *Drosophila* and mammalian models showing that ROS functions upstream to the Akt/Foxo pathway (40, 42–44). Interestingly, inhibiting this pathway failed to rescue the smaller PSC size in *col>Hsc70-4RNAi* (**Figures 3G–L**, quantification in **Figure 3N**), implying that while this pathway mediates non–cell-autonomous signals from the niche that regulate progenitor differentiation into lamellocytes, it does not mediate the cell-autonomous role of Hsc70–4 in controlling PSC cell number through non-apoptotic cell death.

### 2.4 Hsc70–4 depletion in blood cell progenitors causes cell-autonomous lamellocyte differentiation in the lymph gland and their appearance in the circulation

**Figure 4:**
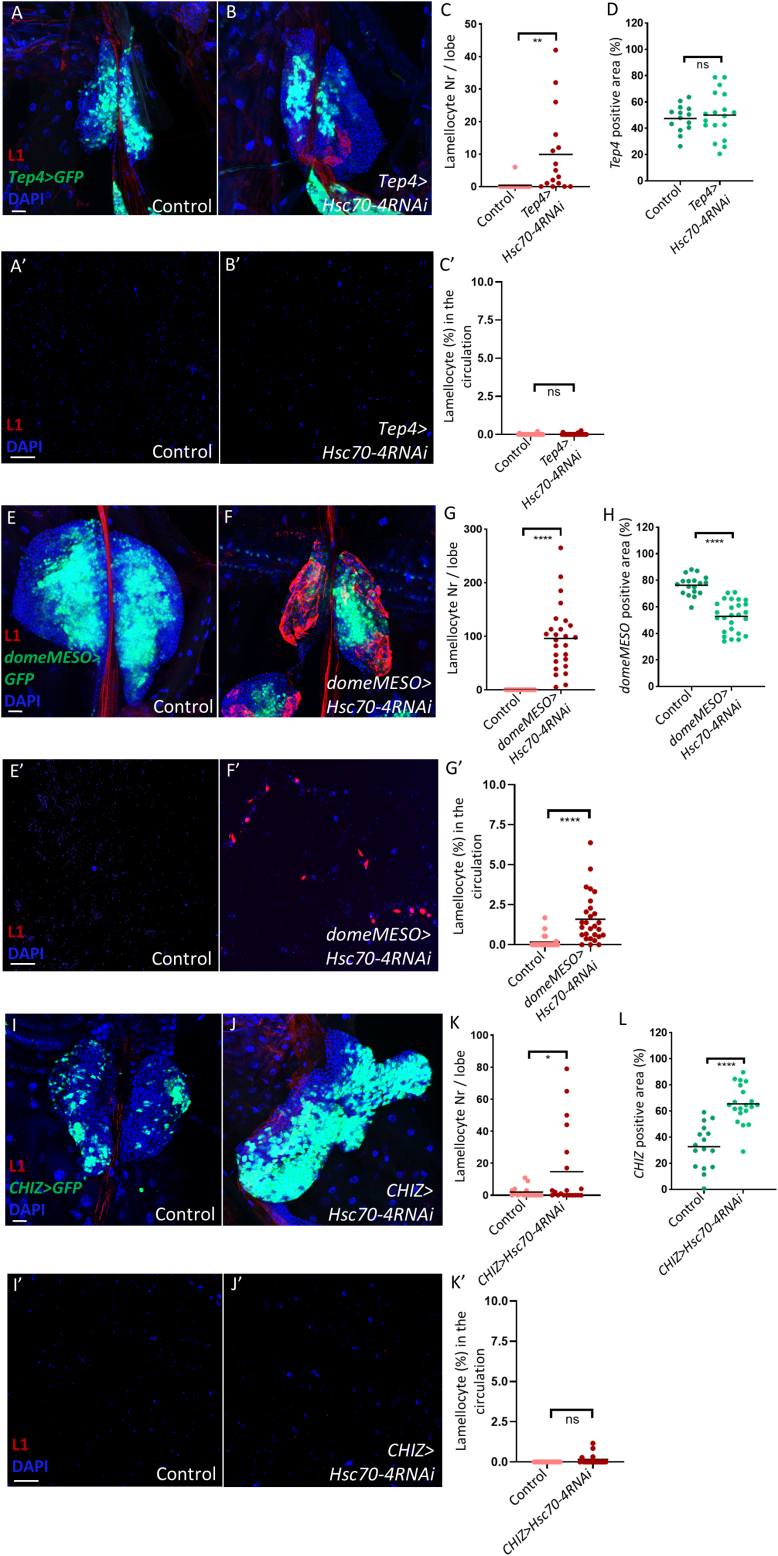
Silencing *Hsc70–4* in blood cell progenitors promotes cell-autonomous lamellocyte differentiation in the lymph gland. **(A, B)** Limited lamellocyte differentiation is observed in the lymph gland when *Hsc70–4* is silenced in core progenitors using the *Tep4-Gal4* driver (*Tep4-Gal4*; *+; UAS-2xEGFP/UAS-Hsc70-4RNAi*) (*n =* 16) **(B)** with no significant effect on *Tep4* positive area compared to the control (*Tep4-Gal4*; *UAS-2xEGFP/+*) (*n =* 16) **(A)** (blue: nuclei, green: core progenitors, red: lamellocytes). *n* refers to the number of lymph gland lobes analyzed. Scale bar: 20 μm. **(C)** A scatter dot plot showing the number of lamellocytes per lymph gland lobe in the genotypes presented in panels **(A, B)**. Each dot on the graph represents one lymph gland lobe. Data were analyzed using two-tailed unpaired Student’s t-test, ** *p* ≤ 0.01. **(D)** A scatter dot plot showing percentage of *Tep4* positive area in larvae from the genotypes presented in panels **(A, B)**. Each dot on the graph represents a PSC from one lymph gland lobe. Data were analyzed using two-tailed unpaired Student’s t-test, *ns*, non-significant. **(A’-B’)** Similarly to the control (*Tep4-Gal4*; *UAS-2xEGFP/+*) (*n =* 24) **(A’)**, significant lamellocyte differentiation is not observed in the circulation when *Hsc70–4* is silenced in lymph gland core progenitors using *Tep4-Gal4* driver (*Tep4-Gal4*; *+; UAS-2xEGFP/UAS-Hsc70-4RNAi*) (*n =* 24) **(B’)** (blue: nuclei, red: lamellocytes). *n* refers to the number of larvae analyzed. Scale bar: 20 μm. **(C’)** A scatter dot plot showing the percentage of lamellocytes in the larval circulation of the genotypes presented in panels **(A’, B’)**. Each dot on the graph represents one larva. Data were analyzed using two-tailed unpaired Student’s t-test, *ns*, non-significant. **(E, F)** Silencing *Hsc70–4* in the MZ of the lymph gland leads to significant lamellocyte differentiation (*domeMESO-Gal4*,*UAS-2xEGFP/UAS-Hsc70-4RNAi*) (*n =* 26) **(F)**, while lamellocytes are normally not detected in the control (*domeMESO-Gal4*,*UAS-2xEGFP/+*) (*n =* 18) **(E)** (blue: nuclei, green: MZ, red: lamellocytes). **(G)** A scatter dot plot showing the number of lamellocytes per lymph gland lobe in the genotypes presented in panels **(E, F)**. Each dot on the graph represents one lymph gland lobe. Data were analyzed using two-tailed unpaired Student’s t-test, ****p ≤ 0.0001. **(H)** A scatter dot plot showing percentage of *domeMESO* positive area in larvae from the genotypes presented in panels **(E, F)**. Each dot on the graph represents a PSC from one lymph gland lobe. Data were analyzed using two-tailed unpaired Student’s t-test, ****p ≤ 0.0001. **(D’, E’)** Silencing *Hsc70–4* in the MZ of the lymph gland leads to significant lamellocyte differentiation in the larval circulation (*domeMESO-Gal4*,*UAS-2xEGFP/UAS-Hsc70-4RNAi*) (*n =* 24) **(E’)**, while lamellocytes are normally not detected in the control (*domeMESO-Gal4*,*UAS-2xEGFP/+*) (*n =* 28) **(D’)** (blue: nuclei, red: lamellocytes). **(F’)** A scatter dot plot showing the percentage of lamellocytes in the larval circulation of the genotypes presented in panels **(D, E)**. Each dot on the graph represents one larva. Data were analyzed using two-tailed unpaired Student’s t-test, ****p ≤ 0.0001. **(I, J)** Limited lamellocyte differentiation is observed in the lymph gland when *Hsc70–4* is silenced in intermediate progenitors using *CHIZ-Gal4* (*CHIZ-Gal4*/*+; UAS-2xEGFP/UAS-Hsc70-4RNAi*) (*n =* 20) **(J)** with significant increase in *CHIZ* positive area compared to the control (*CHIZ-Gal4*/*+*; *UAS-2xEGFP/+*) (*n =* 16) **(I)** (blue: nuclei, green: intermediate progenitors, red: lamellocytes). *n* refers to the number of lymph gland lobes analyzed. Scale bar: 20 μm. **(K)** A scatter dot plot showing the number of lamellocytes per lymph gland lobe in the genotypes presented in panels **(I, J)**. Each dot on the graph represents one lymph gland lobe. Data were analyzed using two-tailed unpaired Student’s t-test, *p ≤ 0.05. **(L)** A scatter dot plot showing percentage of *CHIZ-Gal4* positive area in larvae from the genotypes presented in panels **(I, J)**. Each dot on the graph represents one lymph gland lobe. Data were analyzed using two-tailed unpaired Student’s t-test, ****p ≤ 0.0001. **(I’, J’)** Similarly to the control (*CHIZ-Gal4*/*+*; *UAS-2xEGFP/+*) (*n =* 12) **(J’)**, no significant lamellocyte differentiation is observed in the circulation when *Hsc70–4* is silenced in lymph gland intermediate progenitors using *CHIZ-Gal4* (*CHIZ-Gal4*/*+; UAS-2xEGFP/UAS-Hsc70-4RNAi*) (*n =* 17) **(I’)** (blue: nuclei, red: lamellocytes). *n* refers to the number of larvae analyzed. Scale bar: 20 μm. **(K’)** A scatter dot plot showing the percentage of lamellocytes in the larval circulation of the genotypes presented in panels **(I, J)**. Each dot on the graph represents one larva. Data were analyzed using two-tailed unpaired Student’s t-test, *ns*, non-significant.

After characterizing the non-cell-autonomous role of Hsc70–4 in suppressing lamellocyte fate in the lymph gland PSC cells, we next investigated whether it also functions within progenitors or differentiated hemocytes in a cell-autonomous manner. To assess its role in progenitors, we employed two progenitor-specific drivers: *Tep4-Gal4*, which targets core progenitors, and *domeMESO-Gal4*, which is expressed in both core and distal progenitors (17, 45). Interestingly, silencing *Hsc70–4* using the *Tep4* driver resulted in only limited lamellocyte differentiation in the lymph gland without altering core progenitor size or promoting lamellocyte appearance in the circulation (**Figures 4A, B, A’, B’**, quantification in **Figures 4C, D, C’**). In contrast, Hsc70–4 depletion using *domeMESO-Gal4* triggered extensive lamellocyte differentiation in the lymph gland, accompanied by a significant reduction in MZ size and the emergence of lamellocytes in the hemolymph (**Figures 4E, F, E’, F’**, quantification in **Figures 4G, H, G’**). These results suggest that Hsc70–4 acts cell-autonomously in hemocyte progenitors, particularly within distal progenitors, to restrict their differentiation into lamellocytes. Consistent with findings in the niche, *Hsc70–4* silencing in progenitors also reduced crystal cell differentiation, while leaving plasmatocyte differentiation unaffected (**Supplementary Figures S3A, B, D, E**, quantification in **Supplementary Figures S3C, F**).

Given that *Hsc70–4* knockdown in the niche increased ROS levels, we next asked whether a similar effect occurs in progenitors. Indeed, staining with the ROS-sensitive dye DHE revealed elevated ROS levels in *domeMESO>Hsc70-4RNAi* medullary zones (**Supplementary Figures S3G, H**, quantification in **Supplementary Figure S3I**), suggesting that, similar to the niche, *Hsc70–4* silencing in the MZ triggers cellular stress.

We also explored the role of Hsc70–4 in intermediate progenitors using *CHIZ-Gal4* (46). Silencing *Hsc70–4* in this population led to a significant expansion of intermediate progenitors in the lymph gland, but only weakly promoted lamellocyte differentiation (**Figures 4I, J, I’, J’**, quantification in **Figures 4K, L, K’**). Consistent with this limited differentiation, DHE staining revealed no increase in ROS levels in the IZ of *CHIZ>Hsc70-4RNAi* larvae compared to the control (**Supplementary Figures S3M, N**, quantification in **Supplementary Figure S3O**), likely explaining the minimal lamellocyte differentiation in these animals.

### 2.5 Hsc70–4 depletion in mature blood cells causes their transdifferentiation into lamellocytes in the lymph gland and circulation, and the appearance of melanotic tumors

**Figure 5:**
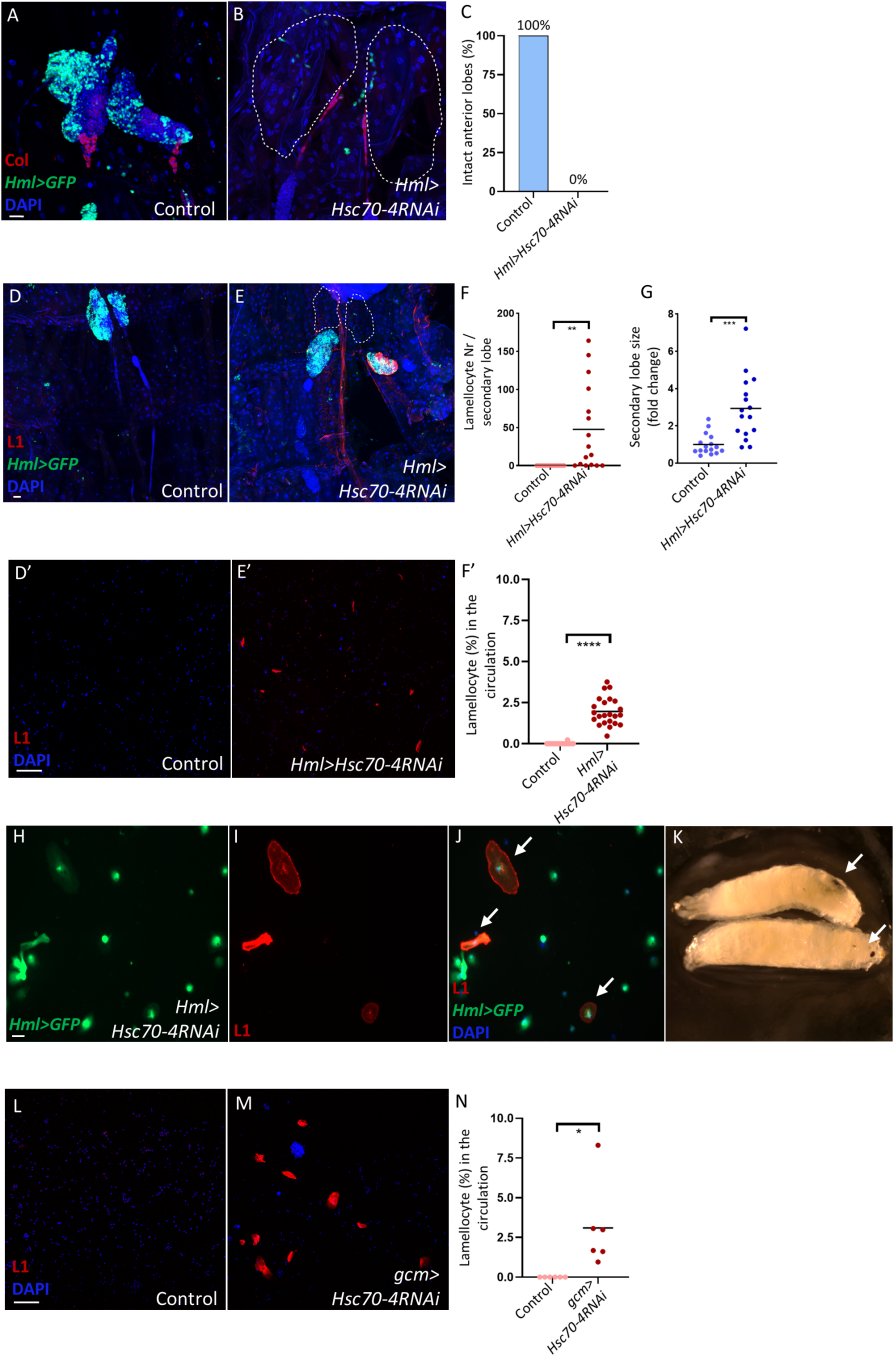
Hsc70–4 depletion in mature blood cells causes their transdifferentiation into lamellocytes in the lymph gland and circulation, and the appearance of melanotic tumors. **(A, B)** Col staining confirming the disintegration in anterior lobes (expected location marked by dashed lines) of *Hml>Hsc70-4RNAi* larvae (*Hml-Gal4*,*UAS-2xEGFP/+; Hml-Gal4*,*UAS-2xEGFP/UAS-Hsc70-4RNAi*) (*n =* 16) **(B)** compared to the control (*Hml-Gal4*,*UAS-2xEGFP/+; Hml-Gal4*,*UAS-2xEGFP/+*) (*n =* 16) **(A)** (blue: nuclei, green: differentiated hemocytes, red: PSC). *n* refers to the number of anterior lymph gland lobes analyzed. Scale bar: 20 μm. **(C)** A bar graph illustrating the percentage of intact anterior lymph gland lobes in the genotypes presented in the panels **(A, B)**. **(D, E)** Silencing *Hsc70–4* in mature hemocytes using *Hml-Gal4* driver causes anterior lobe disintegration (expected location marked by dashed lines) and significant lamellocyte differentiation and enlargement in the secondary lobes of the lymph gland (*Hml-Gal4*,*UAS-2xEGFP/+; Hml-Gal4*,*UAS-2xEGFP/UAS-Hsc70-4RNAi*) (*n =* 16) **(E)** compared to control lymph glands (*Hml-Gal4*,*UAS-2xEGFP/+; Hml-Gal4*,*UAS-2xEGFP/+*) (*n =* 16) **(D)** (blue: nuclei, green: differentiated hemocytes, red: lamellocytes). *n* refers to the number of secondary lymph gland lobes analyzed. Scale bar: 20 μm. **(F)** A scatter dot plot showing the number of lamellocytes per secondary lymph gland lobe in the genotypes presented in panels **(D, E)**. Each dot on the graph represents one secondary lymph gland lobe. Data were analyzed using two-tailed unpaired Student’s t-test, **p ≤ 0.01. **(G)** A scatter dot plot showing secondary lobe size (represented in fold change) from the genotypes presented in panels **(D-E)**. Each dot on the graph represents one secondary lymph gland lobe. Data were analyzed using two-tailed unpaired Student’s t-test, ***p ≤ 0.001. (D’, E’) Silencing *Hsc70–4* in mature hemocytes using *Hml* driver leads to significant lamellocyte differentiation in the circulation (*Hml-Gal4*,*UAS-2xEGFP/+; Hml-Gal4*,*UAS-2xEGFP/UAS-Hsc70-4RNAi*) (*n =* 24) **(E’)** compared to the control (*Hml-Gal4*,*UAS-2xEGFP/+; Hml-Gal4*,*UAS-2xEGFP/+*) (*n =* 23) **(D’)** (blue: nuclei, red: lamellocytes). *n* refers to the number of larvae analyzed. Scale bar: 20 μm. **(F’)** A scatter dot plot showing lamellocyte percentage in the larval circulation of the genotypes presented in panels **(D’, E’)**. Each dot on the graph represents one larva. Data were analyzed using two-tailed unpaired Student’s t-test, ****p ≤ 0.0001. **(H-J)** Immunostaining for the lamellocyte marker L1 reveals the presence of lamellocytes that also express the mature hemocyte marker *Hml* (indicated by arrows) (*n =* 12) (blue: nuclei, green: differentiated hemocytes, red: lamellocytes). *n* refers to the number of larvae analyzed. Scale bar: 20 μm. **(K)** An image showing melanized spots (indicated by arrows) in *Hml>Hsc70-4RNAi* larvae. **(L, M)** Silencing *Hsc70–4* in circulating hemocytes using the *gcm* driver leads to significant lamellocyte differentiation in the circulation (*gcm-Gal4/+; UAS-Hsc70-4RNAi/+*) (*n =* 6) **(M)** compared to the control (*gcm-Gal4/+; +/+*) (*n =* 6) **(L)** (blue: nuclei, red: lamellocytes). *n* refers to the number of larvae analyzed. Scale bar: 20 μm. **(N)** A scatter dot plot showing lamellocyte percentage in the larval circulation of the genotypes presented in panels **(L, M)**. Each dot on the graph represents one larva. Data were analyzed using two-tailed unpaired Student’s t-test, *p ≤ 0.05.

Finally, we explored whether silencing *Hsc70–4* in mature hemocytes would also trigger lamellocyte differentiation. Strikingly, silencing *Hsc70–4* using the *Hml-Gal4* driver (47) caused disintegration of the anterior lobes of the lymph gland, as validated by Col staining, while the secondary lobes showed pronounced enlargement and differentiation of lamellocytes (**Figures 5A-D**, quantification in **Figures 5C, F, G**). Lamellocyte transdifferentiation was also observed in the hemolymph, as evident by the presence of circulating lamellocytes that were also positive for *Hml* (**Figure 5D’, E’, H-J**, quantification in **Figure 5F’**).

Additionally, melanized tumors were observed in *Hml>Hsc70-4RNAi* larvae (**Figure 5K**), a phenotype previously reported in *Hsc70–4* mutants (24). This indicates that this effect specifically results from the lack of Hsc70–4 in mature hemocytes, since its depletion in the PSC or MZ did not cause tumor formation. To confirm that circulating lamellocytes do not originate solely from the lymph gland, we silenced *Hsc70–4* using *gcm-Gal4*, which is specific to circulating hemocytes (48). This also induced lamellocyte differentiation in the circulation, supporting the idea that lamellocytes in *Hml>Hsc70-4RNAi* larvae originate from both the lymph gland and circulating hemocytes.

Altogether, these findings suggest that Hsc70–4 depletion in mature hemocytes disrupts normal differentiation and induces a robust immune response, mimicking conditions such as immune infestation or certain tumorous states, where lymph gland lobes disintegrate and release cells into circulation, leading to cell aggregation and melanotic mass formation (17, 38, 49–52).

## 3 Discussion

*Drosophila melanogaster* has been widely utilized in hematopoietic screens to identify novel regulators of blood cell formation and differentiation (17, 18, 53). In a genetic screen aimed at uncovering genes involved in lamellocyte formation within the *Drosophila* lymph gland, we identified *Hsc70-4* (35, 54). While previous studies have reported that *Hsc70–4* mutant larvae develop melanotic tumors in their hemolymph (24), and that Hsc70–4 plays a role in specifying crystal cell fate in cultured *Drosophila* cells (25, 26), its function within the hematopoietic niche and its impact on lamellocyte differentiation in the lymph gland remained unexplored.

In this study, we uncovered a previously unrecognized role for Hsc70–4 in maintaining the size and function of the hematopoietic niche. Our findings indicate that *Hsc70–4* knockdown significantly reduces niche cell numbers and induces the differentiation of lamellocytes, which are absent under naive conditions, suggesting that Hsc70–4 plays a non-cell-autonomous role in maintaining hemocyte progenitors. Indeed, lamellocyte differentiation was accompanied by a marked decrease in crystal cell numbers in *col>Hsc70-4RNAi* larvae, aligning with our previous research and that of others (27, 55).

To investigate the cause of niche size reduction in *col>Hsc70-4RNAi* animals, we assessed the effects of Hsc70–4 depletion on cell division and cell cycle progression. pH3 staining showed no difference in mitotic activity between *col>Hsc70-4RNAi* and control niches, and overexpression of Myc, a known driver of niche cell proliferation (29), failed to restore niche cell numbers. Similarly, promoting mitosis via *string* (32, 56) overexpression modestly increased PSC size in *col>Hsc70-4RNAi>string* animals, but PSC cell numbers remained significantly lower than in *col>string* controls. These results indicate that reduced niche size is not attributable to impaired proliferation or slowed cell cycle progression. Examination of cell death revealed that Hsc70–4 depletion caused increased 7-AAD staining, which labels dying or dead cells. However, neither the apoptotic marker Dcp-1 nor genetic rescue with the apoptosis inhibitor *p35* supported apoptosis as the underlying cause. FUCCI analysis revealed an almost complete loss of cells in G1, suggesting that G1-phase cells are particularly vulnerable. Together, these findings indicate that Hsc70–4 maintains niche size primarily by preventing non-apoptotic death of G1-phase niche cells.

Next, we explored the mechanism underlying lamellocyte differentiation in *col>Hsc70-4RNAi* animals. Since elevated ROS levels in the PSC are known to promote lamellocyte differentiation via a non-cell-autonomous mechanism (27, 37–39), we asked whether Hsc70–4 depletion in the PSC triggers ROS accumulation. Using two independent PSC drivers (*col-Gal4* and *Antp-Gal4*) and two stress reporters (*gstD-GFP* and *Thor-lacZ*) (39–41), we found that silencing *Hsc70–4* in the niche led to elevated ROS levels. These results are consistent with previous reports that Hsc70–4 loss induces a larval stress response (36). Inhibition of the Akt/Foxo pathway, a key mediator of PSC stress signaling (39), suppressed lamellocyte differentiation but did not reduce ROS levels or restore niche size in *col>Hsc70-4RNAi* animals. These results indicate that Akt/Foxo functions downstream to ROS to mediate non-cell-autonomous lamellocyte differentiation, while the cell-autonomous effect of Hsc70–4 on niche cell survival is independent of this pathway.

Beyond its role in the niche, our data indicate that Hsc70–4 also acts cell-autonomously in hemocyte progenitors to prevent their differentiation into lamellocytes. Silencing *Hsc70–4* in MZ progenitors triggered lamellocyte differentiation, underscoring its requirement for progenitor maintenance. Interestingly, this effect was most pronounced when both core and distal progenitors were targeted, whereas silencing in core progenitors alone had little impact. As we reported similar findings in a recent study involving the progenitor maintenance factor Headcase (27), this suggests that distal progenitors are more plastic and thus more susceptible to fate changes than core progenitors. Consistent with observations in the niche, Hsc70–4 depletion in progenitors reduced crystal cell differentiation, left plasmatocyte differentiation unaffected, and elevated ROS levels. Although we could not test whether the Akt/Foxo pathway also acts downstream to Hsc70–4 in the MZ due to the compromised survival of *domeMESO>Hsc70-4RNAi* adults, prior studies suggest that such a rescue would not be expected, as Akt knockdown in progenitors drives lamellocyte differentiation (57), while Foxo overexpression promotes plasmatocyte and crystal cell fates (58). Supporting this, unlike in the niche, Hsc70–4 depletion in progenitors did not induce *Thor-lacZ* expression (**Supplementary Figures S3J, K**, quantification in **Supplementary Figure S3L**), indicating that progenitors respond to Hsc70–4 loss via mechanisms distinct from those in PSC cells.

We further examined the function of Hsc70–4 in intermediate progenitors using *CHIZ-Gal4* (46). Knockdown of *Hsc70–4* in this population led to a marked expansion of intermediate progenitors but resulted in only minimal lamellocyte differentiation. In line with this, ROS levels remained unchanged in the IZ of *CHIZ>Hsc70-4RNAi* larvae, which explains the weak differentiation response. Together, these findings highlight a cell-autonomous requirement for Hsc70–4 in progenitor maintenance, with distinct responses between core, distal, and intermediate progenitors, further underscoring the heterogeneity of the progenitor pool.

Finally, we investigated whether Hsc70–4 also regulates lamellocyte fate in mature hemocytes. Our findings show that silencing *Hsc70–4* in mature hemocytes triggered a robust immune response, characterized by disintegration of the primary lobes of the lymph glands, lamellocyte transdifferentiation in both the lymph gland and circulation, and the formation of melanotic tumors (17, 51, 59). However, we were unable to assess ROS levels due to the premature disintegration of the primary lobes early in development. Nonetheless, it remains possible that ROS signaling is involved in this context, as previous studies have shown that elevated ROS contribute to the immune response and to lamellocyte transdifferentiation following injury (60).

**Figure 6:**
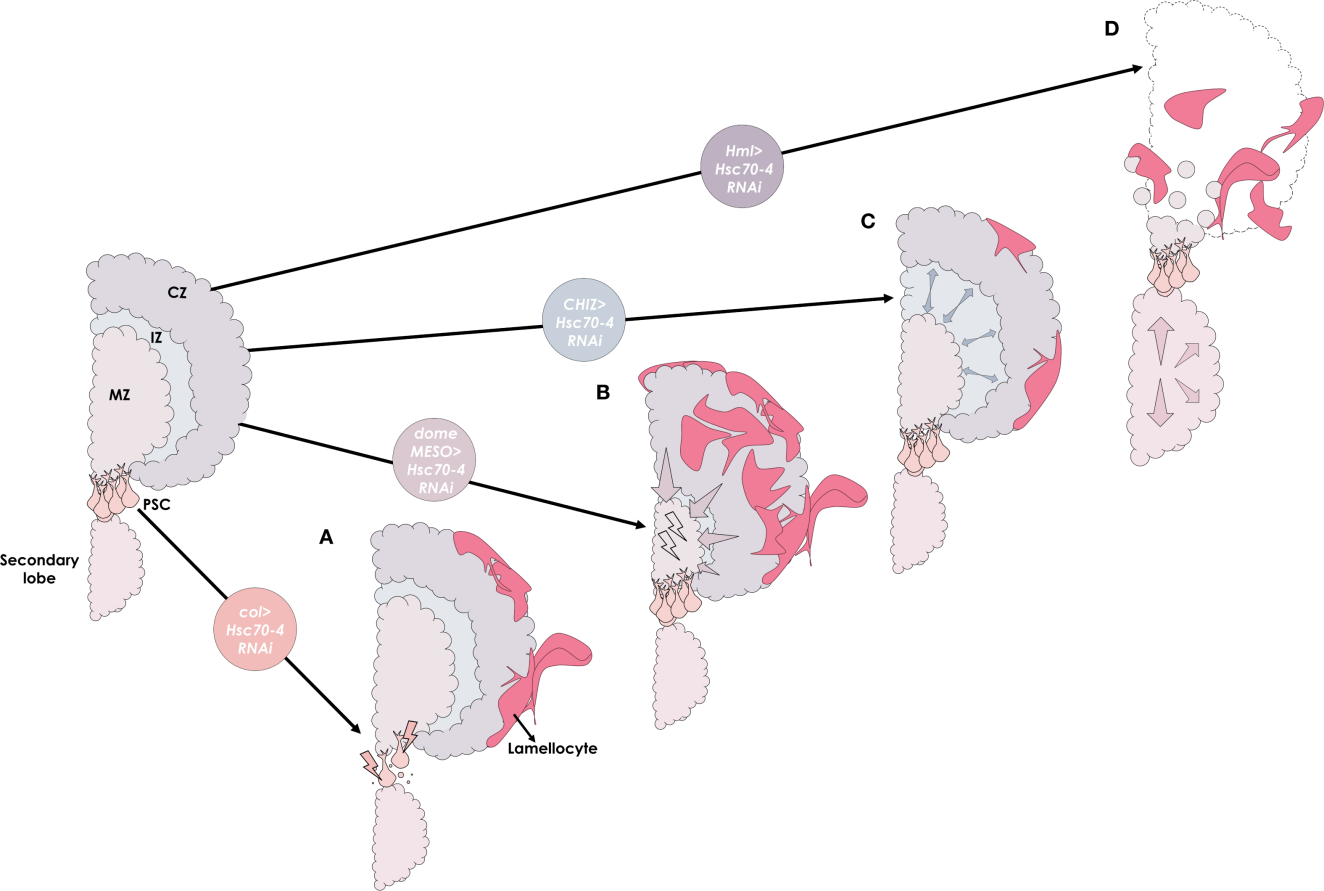
A graphical summary illustrating the distinct roles of the Hsc70–4 chaperone across different domains of the lymph gland. **(A)** Silencing *Hsc70–4* in the hematopoietic niche (PSC) using *col* driver (*col>Hsc70-4RNAi*) reduces niche size through non-apoptotic cell death and non-cell autonomously promotes progenitor differentiation into lamellocytes via ROS accumulation. **(B)** In the medullary zone (MZ), knocking down *Hsc70–4* in progenitors using *domeMESO* driver (*domeMESO>Hsc70-4RNAi*) also causes cellular stress and reduces their number by inducing their differentiation into lamellocytes in a cell-autonomous manner. **(C)** Interestingly, while Hsc70–4 depletion in the intermediate zone (IZ) using *CHIZ* driver (*CHIZ>Hsc70-4RNAi*) leads to only moderate lamellocyte differentiation, it promotes the expansion of the intermediate progenitor pool. **(D)** In the cortical zone (CZ), silencing *Hsc70–4* in mature hemocytes with the *Hml* driver (*Hml>Hsc70-4RNAi*) results in primary lobe disintegration (outlined by dashed lines), secondary lobe enlargement, and transdifferentiation of mature hemocytes into lamellocytes.

This study expands on the diverse roles previously attributed to Hsc70–4 in *Drosophila* (21–23), and demonstrates that Hsc70–4 is essential across all three domains of the larval hematopoietic organ to preserve homeostasis and prevent aberrant differentiation of effector lamellocytes (summarized in **Figure 6**). Our findings also uncover a critical role for Hsc70–4 in the CZ to suppress premature lymph gland dispersal. Since dispersal typically occurs in response to immune challenges such as wasp infestation (55, 61), it would be interesting to explore whether Hsc70–4 levels are dynamically regulated during such stress conditions. Finally, given that HSPA8, the human ortholog of Hsc70-4, has been implicated as a tumor marker in triple-negative breast cancer (62), further investigation into Hsc70-4’s role in cell fate regulation could provide valuable insights into the cellular mechanisms underlying human carcinogenesis.

## 4 Materials and methods

### 4.1 *Drosophila* stocks and maintenance

The following *Drosophila* strains were utilized:

*w^1118^* (BDSC#5905), *UAS-Hsc70-4RNAi#1* (BDSC #28709), *UAS-Hsc70-4RNAi#2* (BDSC#35684), *Pcol85-Gal4,UAS-2xEGFP/SM6b* (6, 27), *Pcol85-Gal4,UAS-2xEGFP/CyO,GFP; UAS-Hsc70-4RNAi#1/TM6Tb* (this study), *UAS-Myc* (BDSC#9674), *UAS-string/CyO,GFP* (BDSC#4777, the *CyO* balancer was changed to *CyO,GFP*), *UAS-p35* (BDSC#5073), *UAS-AktRNAi* (BDSC#31701), *UAS-foxo* (BDSC#9575), *UAS-FlyFUCCI* (BDSC#55122 (31)), *gstD-GFP; Antp-Gal4/Tm6Tb* (generated from combining *gstD-GFP* (a gift from Lolitika Mandal (41) with *Antp-Gal4* (a gift from Gregory D. Longmore (8)), *Thor-lacZ* (BDSC#9558), *domeMESO-Gal4,UAS-2xEGFP/Tm6* (45), *Tep4-Gal4* (a gift from Gregory D. Longmore (17)), *CHIZ-Gal4/CyO,GFP* (a gift from Gregory D. Longmore (46)), *Hml^Turbo^-Gal4* (13), *gcm-gal4* (BDSC#35541), *CHIZ-Gal4,UAS-mGFP* (Banerjee lab). The flies were maintained on standard cornmeal-yeast media at 25 °C, and all crosses were conducted under the same temperature conditions.

### 4.2 Antibodies and reagents

The following primary antibodies were used: mouse anti-L1 and mouse anti-P1 (both used at 1:10 concentration and are a gift from István Andó (63)), mouse anti-Col (1:100, a gift from Michèle Crozatier (6)), mouse anti-LacZ (1:100, DSHB 40-1a), mouse anti-C1 (HC12F6) (a gift from Tina Trenczek), mouse anti-Hnt (1:20, DSHB 1G9), rabbit anti-Dcp1 (1:100, Cell Signaling Technology, CatNo. 9578), rabbit anti-pH3 (1:200, Cell Signaling Technology CatNo. 3642S). Secondary antibodies were: Goat anti-Rabbit Alexa Fluor 568 (1:1000, Thermo Fisher Scientific, CatNo. A-11011), Goat anti-Mouse Alexa Fluor 568 (1:1000, Thermo Fisher Scientific, CatNo. A-11004), Goat anti-Mouse Alexa Fluor Plus 488 (1:1000, Thermo Fisher Scientific, CatNo. A32723), Rabbit anti-Mouse Alexa Fluor 647 (1:1000, Thermo Fisher Scientific, CatNo. A-21239). Nuclei were visualized with DAPI (Sigma-Aldrich).

### 4.3 Immunostaining, imaging, and analysis of lymph glands

Lymph glands were dissected and stained following the protocol detailed in Varga et al. (2019) (64). 7-AAD staining (Cayman Chemical, Cat. No. 11397) was performed at a final concentration of 5 µg/mL. Dissected lymph glands were incubated with the dye for 30 minutes on a shaker, washed three times with 1× PBS, and immediately mounted for imaging under the microscope. DHE (Thermo Fisher Scientific, CatNo. D11347) staining was carried out following the protocol of Evans et al. (2014) (65). Sample numbers provided in the figure legends represent data from three independent experiments for each genotype. For each lymph gland, Z-stacks consisting of 10 slices were captured using the 20× objective (except for images in the panels 4M and 4N which were captured using 10× objective) on Zeiss LSM800 and Zeiss LSM980 confocal microscopes. Images are presented as maximum intensity projections of the Z-stacks, with brightness and contrast adjusted using ImageJ/Fiji (US National Institutes of Health, Bethesda, MD, USA) software. All experimental and control images were captured using identical microscope settings. The number of lamellocytes per lymph gland lobe was manually quantified using the multi-point tool in ImageJ/Fiji by counting DAPI-stained nuclei positive for the L1 antibody. Similarly, PSC cell number (PSC size) was manually determined using the same tool by counting DAPI-stained nuclei positive for *col>GFP* or Col antibody. PSC cells in the G1 (green), S (red), and G2/M (yellow) phases were identified and quantified manually based on their phase-specific colors in the FUCCI system. Additionally, pH3, 7-AAD, Dcp1, and Thor-lacZ positive cells per PSC were manually quantified using the multi-point tool. Crystal cell index was calculated as the ratio of crystal cell number (C1 or Hnt positive cells) to the lymph gland lobe size, measured using the “Analyze > Measure > Area” function in ImageJ/Fiji. The same function was used to measure plasmatocyte (P1 positive), core progenitor (*Tep4>GFP* positive), MZ (*domeMESO>GFP* positive), Intermediate progenitor (*CHIZ>GFP* positive), Mature hemocyte (*Hml>GFP*) or secondary lobe area after the area was selected using the freehand selection tool in ImageJ/Fiji. For measuring fluorescence intensity of *gstD-GFP*, *Thor-LacZ* and DHE markers, the region of interest was selected with the freehand selection tool in ImageJ/Fiji, and mean fluorescence intensity was measured using “Analyze > Measure > Mean Gray Value”. The results were then expressed as fold changes relative to the average fluorescence intensity of the experimental controls.

### 4.4 Immunostaining, imaging, and quantification of circulating hemocytes

Circulating hemocytes from individual larvae were prepared and stained following the protocol described by Varga et al. (2019) (64). Images were captured using the 10× objective (except for images in the panels 5H-5J which were captured using the 40× objective) on a Zeiss Axio Imager Z1 fluorescence and Zeiss LSM800 microscopes. Nuclei were counted automatically using the ‘cellcounter’ macro in ImageJ/Fiji. Lamellocytes (L1-positive cells) were manually counted using the multi-point tool in ImageJ/Fiji, and their percentage relative to the total number of nuclei was calculated. A minimum of 100 nuclei were analyzed per larva.

### 4.5 Imaging of larvae

Whole larvae images were captured using a Flexacam C1 camera on a Leica S9D stereomicroscope with 1× magnification.

### 4.6 Data analysis

All quantitative data analyses and graph generation were performed using GraphPad Prism 8. For comparisons between two groups, a two-tailed unpaired Student’s *t*-test was applied. For datasets involving more than two groups, analysis of variance (ANOVA) followed by Tukey’s test for multiple comparisons was used. Statistical significance was defined as *p* < 0.05, with the following thresholds: *p* ≤ 0.05 (*)*, p ≤ 0.01* (**), *p* ≤ 0.001 (***), *p* ≤ 0.0001 (****), and *ns* indicating non-significance.

### 4.7 Data availability

All relevant data are available within the manuscript and its Supporting Information files. We confirm that all data necessary to replicate the results are included.

## Data Availability

The original contributions presented in the study are included in the article/**Supplementary Material**. Further inquiries can be directed to the corresponding authors.

## Supplementary material

Supplementary Figure 1 Silencing *Hsc70–4* in the niche reduces crystal cell index with no effect on plasmatocyte numbers in the lymph gland. **(A, B)** Knocking down *Hsc70–4* in the PSC (*Pcol85-Gal4,UAS-2xEGFP/+; UAS-Hsc70-4RNAi/+*) significantly decreases the crystal cell index (average = 2.6, *n =* 28) **(B)**, compared to the control (*Pcol85-Gal4,UAS-2xEGFP/+*) (average = 5.8, *n =* 12) **(A)** (blue: nuclei, green: PSC, red: crystal cells). *n* indicates the number of lymph gland lobes examined. Scale bar: 20 μm. **(C)** A scatter dot plot showing the crystal cell index quantified from the genotypes in the panels **(A, B)**. Each dot on the graph represents a single lymph gland lobe. Data were analyzed using two-tailed unpaired Student’s t-test, ** *p* ≤ 0.01. **(D, E)** Knocking down *Hsc70–4* in the PSC (*Pcol85-Gal4,UAS-2xEGFP/+; UAS-Hsc70-4RNAi/+*) (average = 59.15%, *n =* 20) **(E)** has no impact on the percentage of P1-positive (plasmatocyte) area per anterior lobe compared to the control (*Pcol85-Gal4,UAS-2xEGFP/+*) (average = 60.36%, *n =* 14) **(D)** (blue: nuclei, green: PSC, red: plasmatocytes). Scale bar: 20 μm. **(F)** A scatter dot plot illustrating the percentage of P1 positive (plasmatocyte) area per anterior lobe based on the genotypes presented in the panels **(D, E)**. Each dot on the graph represents a single lymph gland lobe. Data were analyzed using two-tailed unpaired Student’s t-test, *ns*, non-significant. **(G, H)** Silencing *Hsc70–4* using another PSC-specific driver, *Antp-Gal4*, causes smaller niche size (outlined by dashed lines, based on Col staining) (*gstD-GFP/+; Antp-Gal4/UAS-Hsc70-4RNAi*) (*n =* 12) **(H)**, compared to the control (*gstD-GFP/+; Antp-Gal4/+*) (*n =* 20) **(G)** (blue: nuclei, red: PSC). *n* indicates the number of lymph gland lobes examined. Scale bar: 20 μm. **(I)** A scatter dot plot showing PSC cell number in larvae from the genotypes presented in panels **(G, H)**. Each dot on the graph represents a PSC from one lymph gland lobe. Data were analyzed using two-tailed unpaired Student’s t-test, **** *p* ≤ 0.0001.

Supplementary Figure 2 Knocking down the Akt/Foxo pathway does not rescue the increase in ROS in the PSC upon *Hsc70–4* silencing. **(A, B)** Knocking down *Hsc70–4* in the PSC (*Pcol85-Gal4,UAS-2xEGFP/+; UAS-Hsc70-4RNAi/+*) significantly increases ROS levels in the niche (outlined by dashed lines, based on *col>GFP* expression) as assayed by DHE (*n =* 7) **(B)** compared to the control (*Pcol85-Gal4,UAS-2xEGFP/+; +/+*) (*n =* 11) **(A)**. **(C, D)** inhibiting the Akt/Foxo pathway through silencing *Akt* (*Pcol85-Gal4,UAS-2xEGFP/+; UAS-Hsc70-4RNAi/UAS-AktRNAi*) (*n =* 8) **(C)** or overexpression of *foxo* (*Pcol85-Gal4,UAS-2xEGFP/UAS-foxo; UAS-Hsc70-4RNAi/+*) (*n =* 10) does not rescue the increase in ROS levels in the niche (outlined by dashed lines, based on *col>GFP* expression) of *col>Hsc70-4RNAi* larvae. **(E)** A scatter dot plot showing the mean fluorescence intensity of DHE in the PSC represented in fold change quantified from the genotypes in the panels **(A-D)**. Each dot on the graph represents a PSC from a single lobe. Data were analyzed using ANOVA with Tukey’s test for multiple comparisons, ****p* ≤ 0.001, *ns*, non-significant.

Supplementary Figure 3 Silencing *Hsc70–4* in the MZ reduces crystal cell index with no effect on plasmatocyte numbers in the lymph gland. **(A, B)** Knocking down *Hsc70–4* in the MZ (+/+; *domeMESO-Gal4*,*UAS-2xEGFP/UAS-Hsc70-4RNAi*) significantly decreases the crystal cell index fold change (*n =* 14) **(B)**, compared to the control (*+/+; domeMESO-Gal4*,*UAS-2xEGFP/+*) (*n =* 8) **(A)** (blue: nuclei, red: crystal cells). *n* indicates the number of lymph gland lobes examined. Scale bar: 20 μm. **(C)** A scatter dot plot showing the crystal cell index represented in fold change quantified from the genotypes in the panels **(A, B)**. Each dot on the graph represents a single lymph gland lobe. Data were analyzed using two-tailed unpaired Student’s t-test, *** *p* ≤ 0.001. **(D, E)** Knocking down *Hsc70–4* in the MZ (+/+; *domeMESO-Gal4*,*UAS-2xEGFP/UAS-Hsc70-4RNAi*) has no impact on the percentage of P1-positive (plasmatocyte) area per anterior lobe (*n =* 8) **(E)** compared to the control (*+/+; domeMESO-Gal4*,*UAS-2xEGFP/+*) (*n =* 12) **(D)** (blue: nuclei, red: plasmatocytes). Scale bar: 20 μm. **(F)** A scatter dot plot illustrating the percentage of P1 positive (plasmatocyte) area per anterior lobe based on the genotypes presented in the panels **(D, E)**. Each dot on the graph represents a single lymph gland lobe. Data were analyzed using two-tailed unpaired Student’s t-test, *ns*, non-significant. **(G, H)** Knocking down *Hsc70–4* in the MZ (+/+; *domeMESO-Gal4*,*UAS-2xEGFP/UAS-Hsc70-4RNAi*) significantly increases the fold change of DHE fluorescence intensity in the MZ (outlined by dashed lines, based on *domeMESO>GFP* expression) (*n =* 16) **(H)** compared to the control (*+/+; domeMESO-Gal4*,*UAS-2xEGFP/+*) (*n =* 16) **(G)** (red: DHE). *n* indicates the number of lymph gland lobes examined. Scale bar: 20 μm. **(I)** A scatter dot plot showing DHE fluorescence intensity represented in fold change quantified from the genotypes in the panels **(G-H)**. Each dot on the graph represents a single lymph gland lobe. Data were analyzed using two-tailed unpaired Student’s t-test, **** *p* ≤ 0.0001. **(J, K)** Knocking down *Hsc70–4* in the MZ (+/+; *domeMESO-Gal4*,*UAS-2xEGFP/UAS-Hsc70-4RNAi*) does not change *Thor-LacZ* fluorescence intensity in this zone (outlined by dashed lines, based on *domeMESO>GFP* expression) (*n =* 10) **(K)** compared to the control (*+/+; domeMESO-Gal4*,*UAS-2xEGFP*) (*n =* 9) **(J)** (red: Thor-lacZ). *n* indicates the number of lymph gland lobes examined. Scale bar: 20 μm. **(L)** A scatter dot plot showing DHE fluorescence intensity represented in fold change quantified from the genotypes in the panels **(J, K)**. Each dot on the graph represents a single lymph gland lobe. Data were analyzed using two-tailed unpaired Student’s t-test, *ns*, non-significant. **(M, N)** Knocking down *Hsc70–4* in the IZ (outlined by dashed lines, based on *CHIZ>GFP* expression) (*CHIZ-Gal4,UAS-mGFP*/*+; UAS-Hsc70-4RNAi/+*) does not increase the fold change of DHE fluorescence intensity in the IZ (*n =* 18) **(N)** compared to the control (*CHIZ-Gal4,UAS-mGFP*/*+; +/+)* (*n =* 16) **(M)** (red: DHE). *n* indicates the number of lymph gland lobes examined. Scale bar: 20 μm. **(O)** A scatter dot plot showing DHE fluorescence intensity represented in fold change quantified from the genotypes in the panels **(M, N)**. Each dot on the graph represents a single lymph gland lobe. Data were analyzed using two-tailed unpaired Student’s t-test, *ns*, non-significant.
