# Supplementary figures and images for: A novel role for Hsc70–4 in blood cell differentiation in Drosophila
Source: Front Immunol. 2025 Oct 8;16:1641695. doi: 10.3389/fimmu.2025.1641695 (PMC12540110; doi:10.3389/fimmu.2025.1641695)

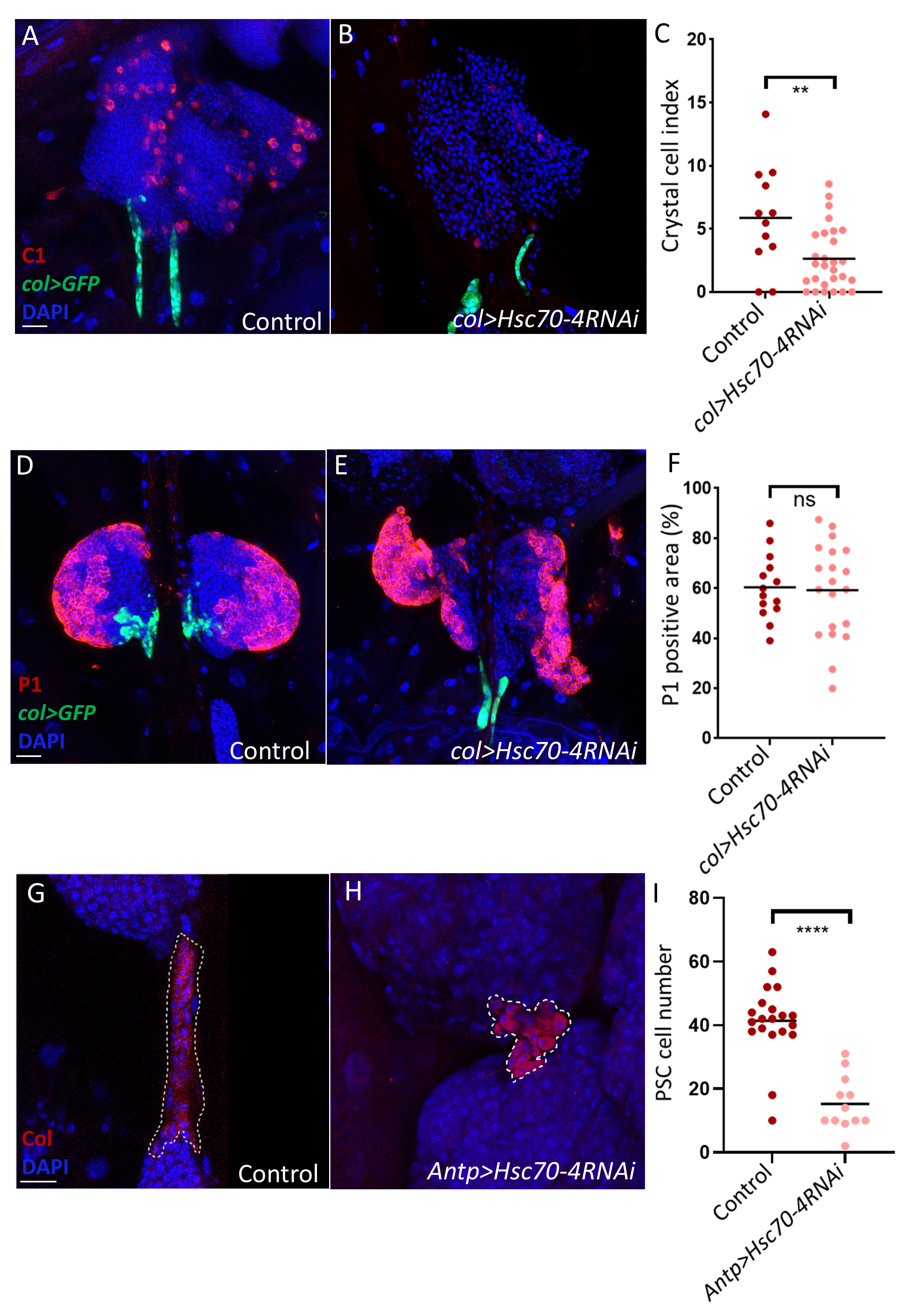

Supplement: Supplementary Figure 1 — Silencing Hsc70–4 in the niche reduces crystal cell index with no effect on plasmatocyte numbers in the lymph gland. (A, B) Knocking down Hsc70–4 in the PSC (Pcol85-Gal4,UAS-2xEGFP/+; UAS-Hsc70-4RNAi/+) significantly decreases the crystal cell index (average = 2.6, n = 28) (B), compared to the control (Pcol85-Gal4,UAS-2xEGFP/+) (average = 5.8, n = 12) (A) (blue: nuclei, green: PSC, red: crystal cells). n indicates the number of lymph gland lobes examined. Scale bar: 20 μm. (C) A scatter dot plot showing the crystal cell index quantified from the genotypes in the panels (A, B). Each dot on the graph represents a single lymph gland lobe. Data were analyzed using two-tailed unpaired Student’s t-test, ** p ≤ 0.01. (D, E) Knocking down Hsc70–4 in the PSC (Pcol85-Gal4,UAS-2xEGFP/+; UAS-Hsc70-4RNAi/+) (average = 59.15%, n = 20) (E) has no impact on the percentage of P1-positive (plasmatocyte) area per anterior lobe compared to the control (Pcol85-Gal4,UAS-2xEGFP/+) (average = 60.36%, n = 14) (D) (blue: nuclei, green: PSC, red: plasmatocytes). Scale bar: 20 μm. (F) A scatter dot plot illustrating the percentage of P1 positive (plasmatocyte) area per anterior lobe based on the genotypes presented in the panels (D, E). Each dot on the graph represents a single lymph gland lobe. Data were analyzed using two-tailed unpaired Student’s t-test, ns, non-significant. (G, H) Silencing Hsc70–4 using another PSC-specific driver, Antp-Gal4, causes smaller niche size (outlined by dashed lines, based on Col staining) (gstD-GFP/+; Antp-Gal4/UAS-Hsc70-4RNAi) (n = 12) (H), compared to the control (gstD-GFP/+; Antp-Gal4/+) (n = 20) (G) (blue: nuclei, red: PSC). n indicates the number of lymph gland lobes examined. Scale bar: 20 μm. (I) A scatter dot plot showing PSC cell number in larvae from the genotypes presented in panels (G, H). Each dot on the graph represents a PSC from one lymph gland lobe. Data were analyzed using two-tailed unpaired Student’s t-test, **** p ≤ 0.0001. [file Image1.tif]

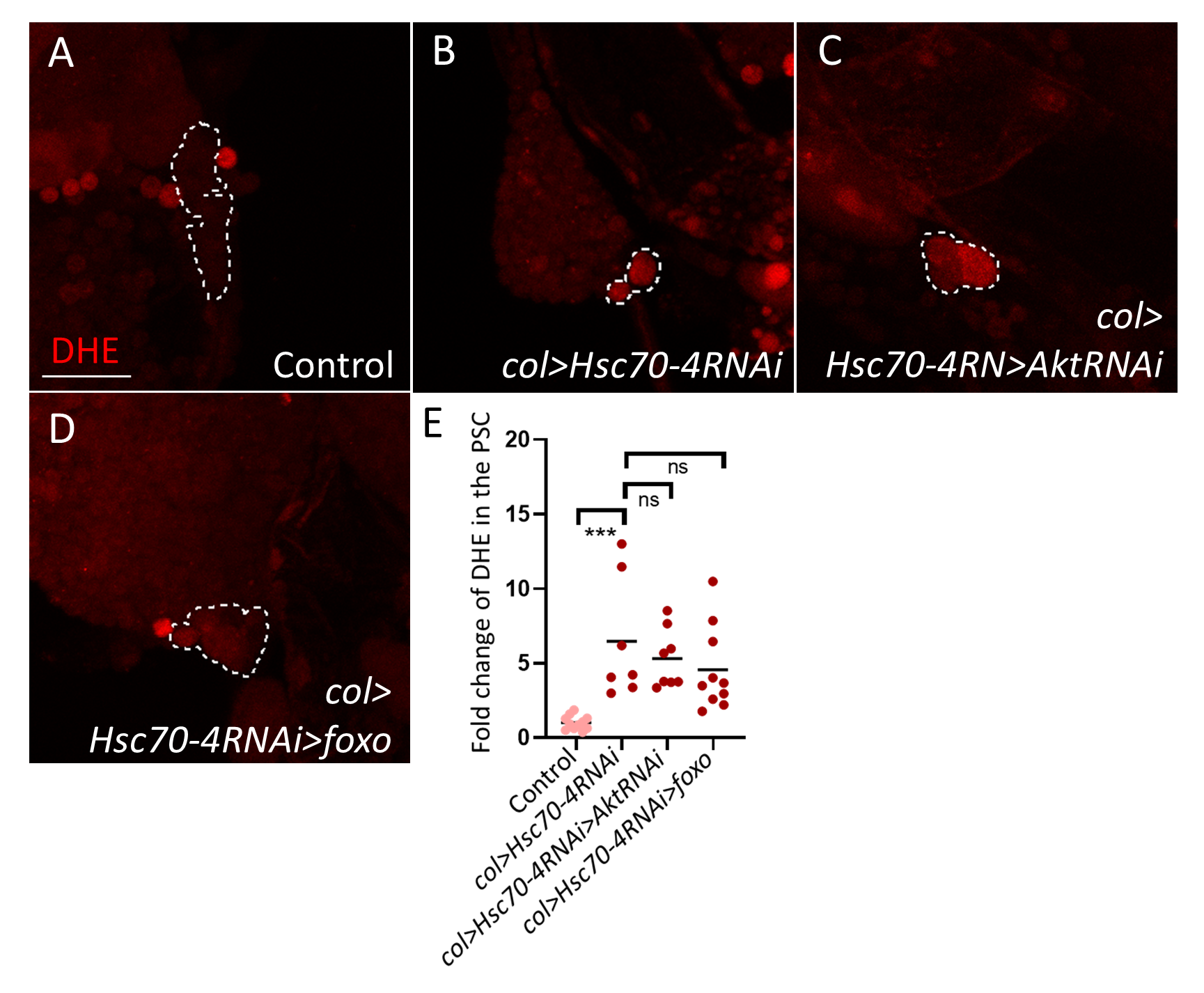

Supplement: Supplementary Figure 2 — Knocking down the Akt/Foxo pathway does not rescue the increase in ROS in the PSC upon Hsc70–4 silencing. (A, B) Knocking down Hsc70–4 in the PSC (Pcol85-Gal4,UAS-2xEGFP/+; UAS-Hsc70-4RNAi/+) significantly increases ROS levels in the niche (outlined by dashed lines, based on col>GFP expression) as assayed by DHE (n = 7) (B) compared to the control (Pcol85-Gal4,UAS-2xEGFP/+; +/+) (n = 11) (A). (C, D) inhibiting the Akt/Foxo pathway through silencing Akt (Pcol85-Gal4,UAS-2xEGFP/+; UAS-Hsc70-4RNAi/UAS-AktRNAi) (n = 8) (C) or overexpression of foxo (Pcol85-Gal4,UAS-2xEGFP/UAS-foxo; UAS-Hsc70-4RNAi/+) (n = 10) does not rescue the increase in ROS levels in the niche (outlined by dashed lines, based on col>GFP expression) of col>Hsc70-4RNAi larvae. (E) A scatter dot plot showing the mean fluorescence intensity of DHE in the PSC represented in fold change quantified from the genotypes in the panels (A-D). Each dot on the graph represents a PSC from a single lobe. Data were analyzed using ANOVA with Tukey’s test for multiple comparisons, ***p ≤ 0.001, ns, non-significant. [file Image2.tif]

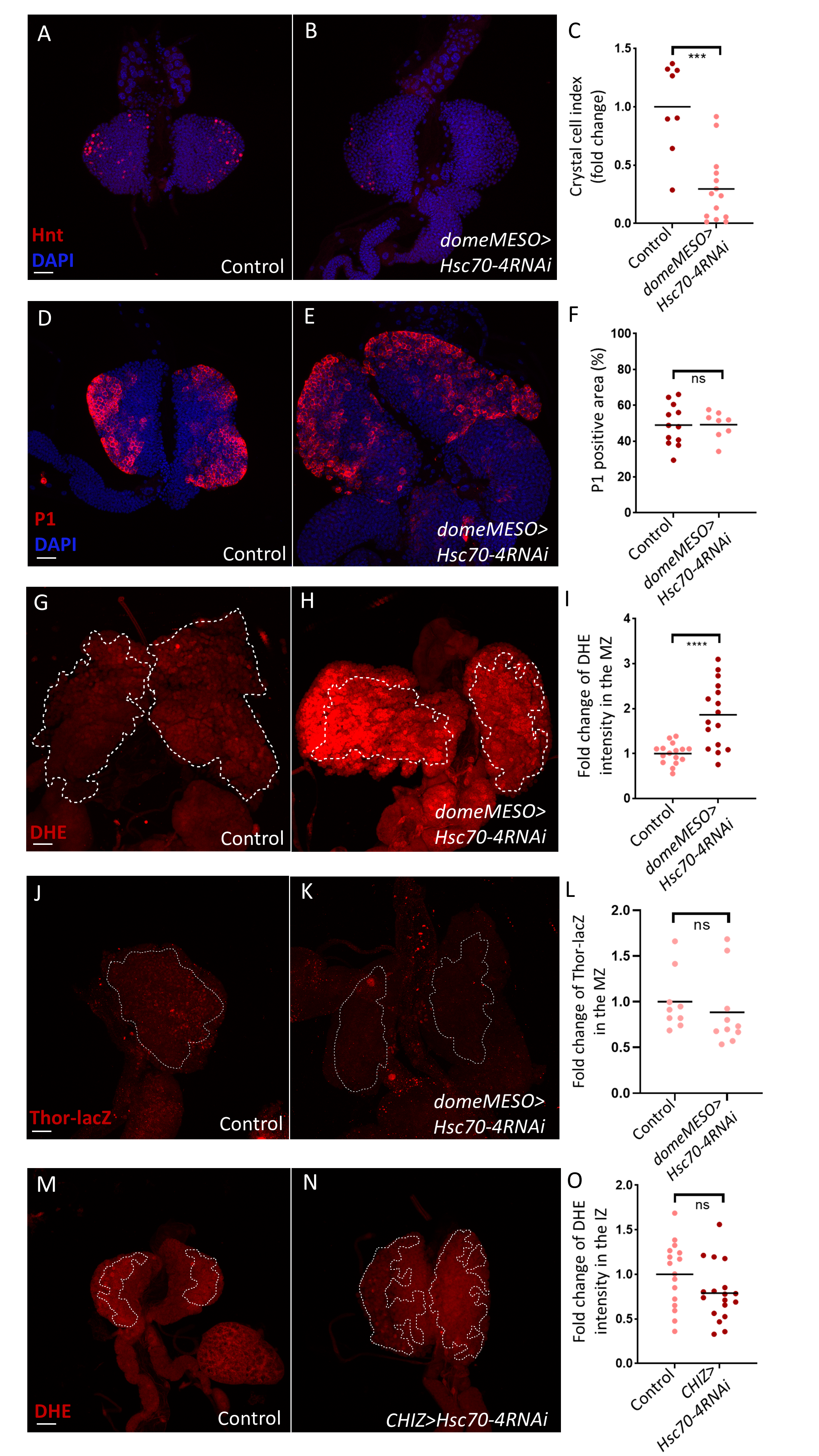

Supplement: Supplementary Figure 3 — Silencing Hsc70–4 in the MZ reduces crystal cell index with no effect on plasmatocyte numbers in the lymph gland. (A, B) Knocking down Hsc70–4 in the MZ (+/+; domeMESO-Gal4,UAS-2xEGFP/UAS-Hsc70-4RNAi) significantly decreases the crystal cell index fold change (n = 14) (B), compared to the control (+/+; domeMESO-Gal4,UAS-2xEGFP/+) (n = 8) (A) (blue: nuclei, red: crystal cells). n indicates the number of lymph gland lobes examined. Scale bar: 20 μm. (C) A scatter dot plot showing the crystal cell index represented in fold change quantified from the genotypes in the panels (A, B). Each dot on the graph represents a single lymph gland lobe. Data were analyzed using two-tailed unpaired Student’s t-test, *** p ≤ 0.001. (D, E) Knocking down Hsc70–4 in the MZ (+/+; domeMESO-Gal4,UAS-2xEGFP/UAS-Hsc70-4RNAi) has no impact on the percentage of P1-positive (plasmatocyte) area per anterior lobe (n = 8) (E) compared to the control (+/+; domeMESO-Gal4,UAS-2xEGFP/+) (n = 12) (D) (blue: nuclei, red: plasmatocytes). Scale bar: 20 μm. (F) A scatter dot plot illustrating the percentage of P1 positive (plasmatocyte) area per anterior lobe based on the genotypes presented in the panels (D, E). Each dot on the graph represents a single lymph gland lobe. Data were analyzed using two-tailed unpaired Student’s t-test, ns, non-significant. (G, H) Knocking down Hsc70–4 in the MZ (+/+; domeMESO-Gal4,UAS-2xEGFP/UAS-Hsc70-4RNAi) significantly increases the fold change of DHE fluorescence intensity in the MZ (outlined by dashed lines, based on domeMESO>GFP expression) (n = 16) (H) compared to the control (+/+; domeMESO-Gal4,UAS-2xEGFP/+) (n = 16) (G) (red: DHE). n indicates the number of lymph gland lobes examined. Scale bar: 20 μm. (I) A scatter dot plot showing DHE fluorescence intensity represented in fold change quantified from the genotypes in the panels (G-H). Each dot on the graph represents a single lymph gland lobe. Data were analyzed using two-tailed unpaired Student’s t-test, **** p ≤ 0 [file Image3.tif]
